# Pop-in behavior and elastic-to-plastic transition of polycrystalline pure iron during sharp nanoindentation

**DOI:** 10.1038/s41598-019-51644-5

**Published:** 2019-10-25

**Authors:** Fabian Pöhl

**Affiliations:** Ruhr-Universität Bochum, Chair of Materials Technology, Bochum, 44780 Germany

**Keywords:** Materials science, Engineering

## Abstract

This study analyzes the elastic-to-plastic transition during nanoindentation of polycrystalline iron. We conduct nanoindentation (Berkovich indenter) experiments and electron backscatter diffraction analysis to investigate the initiation of plasticity by the appearance of the pop-in phenomenon in the loading curves. Numerous load–displacement curves are statistically analyzed to identify the occurrence of pop-ins. A first pop-in can result from plasticity initiation caused by homogeneous dislocation nucleation and requires shear stresses in the range of the theoretical strength of a defect-free iron crystal. The results also show that plasticity initiation in volumes with preexisting dislocations is significantly affected by small amounts of interstitially dissolved atoms (such as carbon) that are segregated into the stress fields of dislocations, impeding their mobility. Another strong influence on the pop-in behavior is grain boundaries, which can lead to large pop-ins at relatively high indentation loads. The pop-in behavior appears to be a statistical process affected by interstitial atoms, dislocation density, grain boundaries, and surface roughness. No effect of the crystallographic orientation on the pop-in behavior can be observed.

## Introduction

Indentation testing, particularly nanoindentation, is widely used for local mechanical characterization of materials and single phases in multiphase materials. It enables the determination of important mechanical parameters such as the hardness, Young’s modulus, or indentation energy parameters and contributes greatly to the understanding of the mechanical behavior on the nanometer length scale^1,2^. When smaller length scales are investigated, unique deformation phenomena appear. One phenomenon that is sometimes observed at shallow indentation depths is a sudden displacement burst appearing as a plateau in the loading curve (so-called pop-in). There are several possible explanations for the occurrence of pop-ins. According to the literature, pop-ins have been observed as a result of phase transformation^3,4^, fracture with crack initiation and propagation^5^, or homogeneous dislocation nucleation^6–10^. In ductile metallic materials without mechanically induced phase transformations, the latter mechanism is highly important. The initial contact of a sharp indenter such as the Berkovich indenter with a metallic material is elastic due to the tip rounding of the indenter. Hence, it is assumed that the initial contact can be described as the contact of a spherical body and an elastic half-space. Thus, the Hertz contact theory gives the following relationship (Eq. 1) between the applied load *P* and the resulting indentation depth *h*^11^.

1 $$P=\frac{4}{3}{E}_{{\rm{r}}}\sqrt{R}{h}^{\frac{3}{2}}$$

The parameter *R* represents the radius of a spherical indenter, and *E*_r_ is the reduced modulus of the contact, which is given by Eq. 2.

2 $$\frac{1}{{E}_{{\rm{r}}}}=\frac{1-{\nu }_{i}^{2}}{{E}_{{\rm{i}}}}+\frac{1-{\nu }_{{\rm{s}}}^{2}}{{E}_{{\rm{s}}}}$$

Here, *E* is the Young’s modulus, and *ν* is the Poisson’s ratio; the superscript letters i and s indicate the values for the indenter and specimen, respectively. The analytical solution given by Hertz also enables the calculation of the induced shear stress in the material under the spherical indenter tip according to Eq. 3 ^11^.

3 $$\tau =0.31{(\frac{6}{{\pi }^{3}}\frac{P{E}_{{\rm{r}}}^{2}}{{R}^{2}})}^{\frac{1}{3}}$$

These equations are often used to analyze a first pop-in, which is largely the result of plasticity initiation by dislocation nucleation in ductile metals. It has been found that very high shear stresses in the range of the theoretical strength are necessary for plasticity initiation, which is consistent with homogeneous dislocation nucleation in a defect-free volume^10^. These findings are also well supported by molecular dynamics (MD) simulations of face-centered cubic (fcc) metals^8^. However, several features and influencing factors such as the crystallographic orientation, interstitial atoms in body-centered cubic (bcc) metals, surface roughness, and preexisting dislocation density are still not fully understood. In this study, we analyzed a possible correlation between the crystallographic orientation and the occurrence of pop-ins in pure polycrystalline iron as a model material with bcc lattice structure. Furthermore, the effect of interstitial atoms such as carbon and nitrogen on the pop-in behavior were investigated, as small amounts of these elements appeared in the pure iron. The surface conditions, that is, the surface roughness and dislocation density, were also altered in the analysis to study their effect on the pop-in characteristics.

## Materials and Methods

### Materials

Polycrystalline pure iron with a purity of 99.8% was analyzed in a recrystallized condition to ensure a large grain size and low defect density. Table 1 summarizes the chemical composition measured by optical emission spectroscopy. The iron contains carbon and nitrogen at 0.007 mass% each. Specimen preparation for microstructure analysis and nanoindentation testing included grinding with SiC paper and polishing with diamond suspension to a final polishing step with an average diamond grain size of 1 *μ*m. To minimize surface hardening and produce a smooth surface, surface finishing was performed using a colloidal fine suspension containing amorphous not-crystallizing silicates 0.02 *μ*m in size (MasterMet II).

**Table 1:** Chemical composition of the investigated iron in mass% measured by optical emission spectroscopy.

C	N	Mn	Cr	Ni	Fe
0.007	0.007	0.042	0.013	0.013	bal.

### Nanoindentation and tensile testing

**Figure 1:**
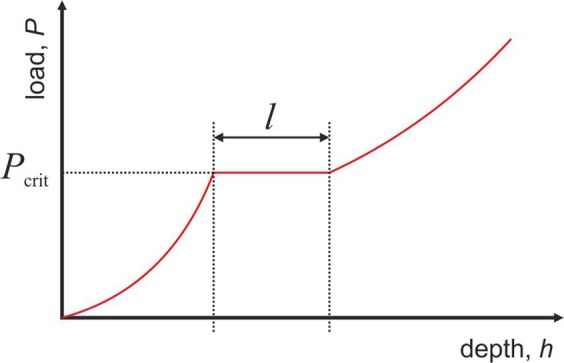
Schematic illustration of a pop-in and its description in terms of the critical load *P*_crit_ and length *l*.

Nanoindentation experiments were conducted using a nanoindenter (iMicro, Nanomechanics) equipped with a diamond Berkovich tip. The loading and unloading rate were constant at 0.2 s^−1^, and the maximum load was 10 mN. The obtained load–displacement curves (*P*–*h* curves) were evaluated to identify the occurrence of pop-ins. The number of pop-ins with the critical pop-in initiation load *P*_crit_ and pop-in length *l* (Fig. 1) was determined.

Tensile testing was conducted using a Z100 device from Zwick at a constant strain rate of 3 · 10^−3^ s^−1^ to obtain the stress–strain curve. Strain gauges were used to accurately determine the Young’s modulus. Tensile specimens had a spherical geometry with a radius of 5 mm in the gauge length.

### Scanning electron microscopy

A scanning electron microscope (Mira 3, Tescan) with an electron backscatter diffraction (EBSD) detector was used to characterize the microstructure and to analyze the crystallographic orientation of the specimen. We used an Oxford EBSD detector (NordlysNano) with an acceleration voltage of 15 kV, a working distance of 15 mm, and a scan step size of 0.2 *μ*m.

## Results and Discussion

**Figure 2:**
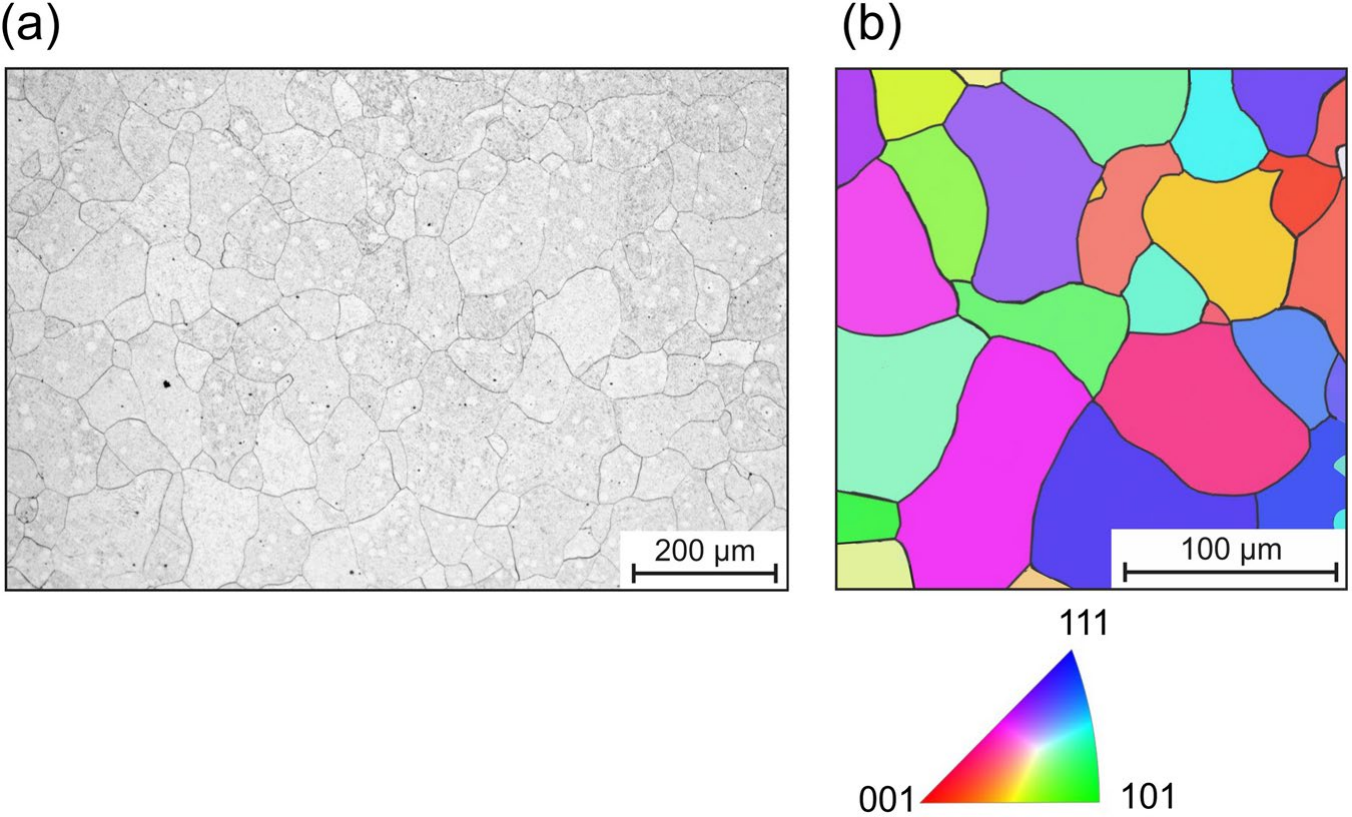
Microstructure of the pure iron: (**a**) SEM image after etching and (**b**) EBSD image (IPFZ map).

**Figure 3:**
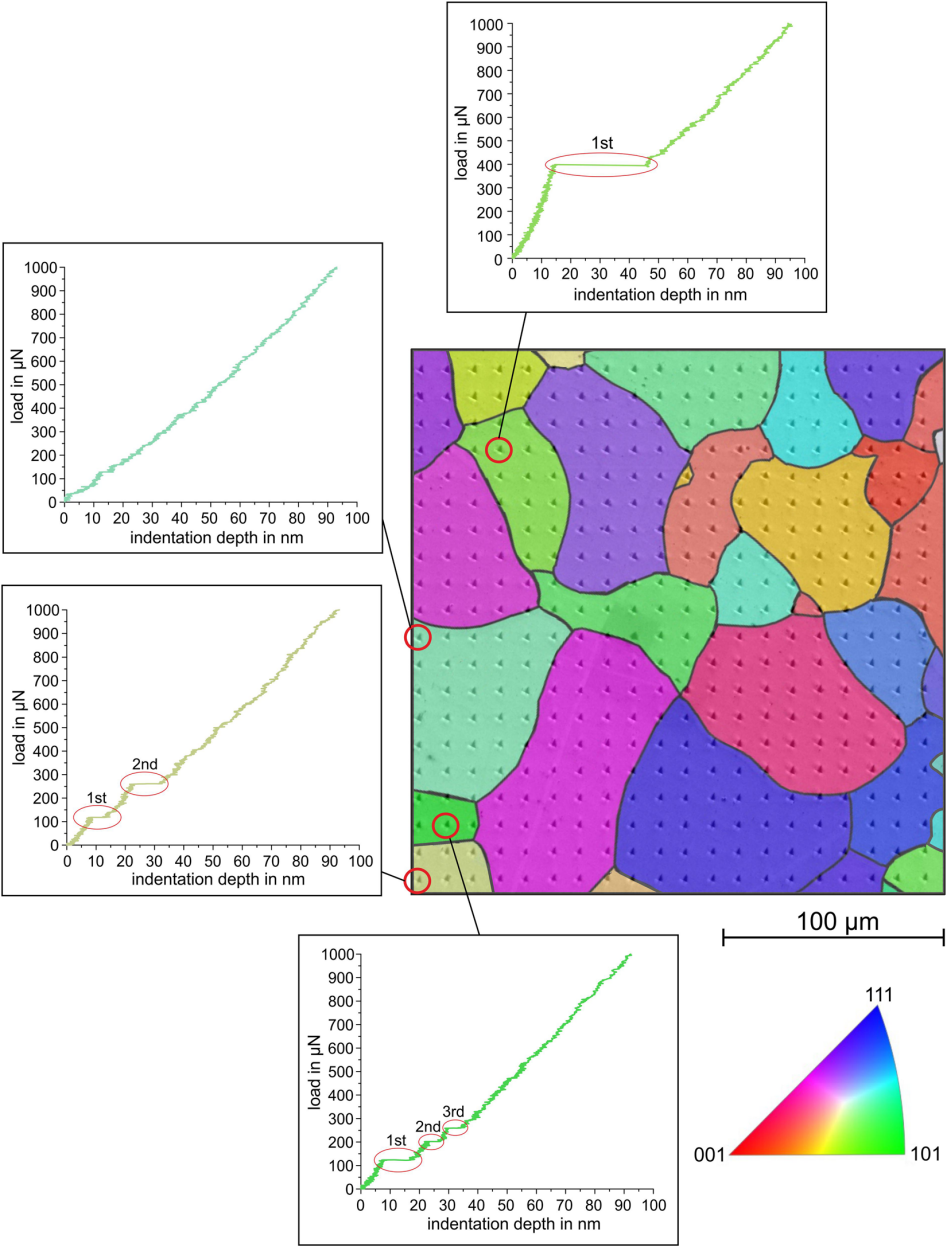
Nanoindentation array in polycrystalline pure iron with superimposed EBSD analysis and selected loading curves with zero, one, two, and three pop-ins.

The microstructure of the polycrystalline iron is shown in Fig. 2. It has globular grains with an average size of approximately 50 *μ*m. Figure 3 shows an array of nanoindentations superimposed on the results of an EBSD analysis of this location. The figure also contains selected loading curves showing zero, one, two, and even three pop-ins. A total of 400 indentations were analyzed to identify the occurrence of pop-ins. Here, we analyzed the loading curve up to a load of 1000 *μ*N, although isolated pop-ins were observed even at larger loads. As described in the introduction, the occurrence of a first pop-in can be attributed to the transition from purely elastic behavior to the onset of additional plastic deformation.

**Figure 4:**
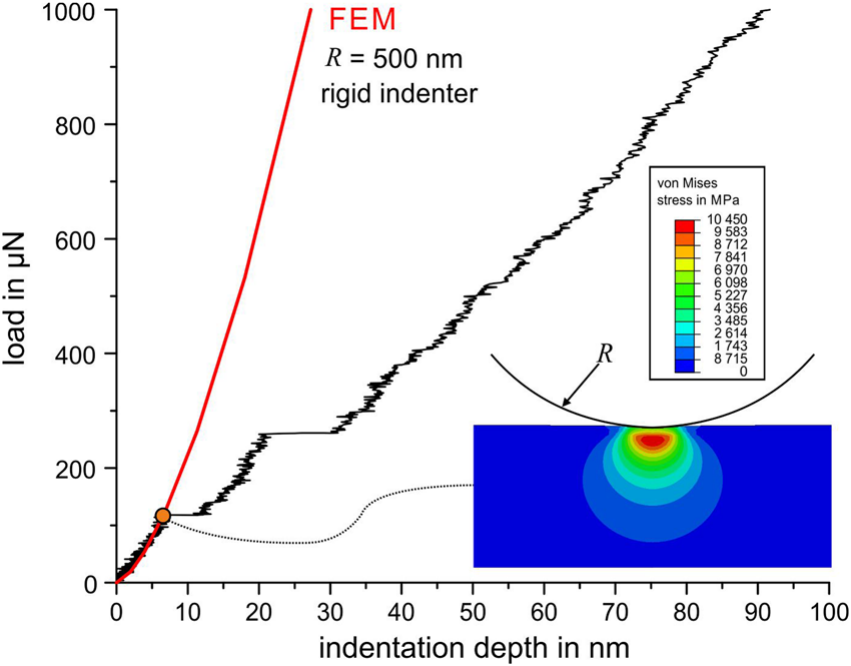
Measured loading curve with two pop-ins, with analytically (Hertz) and numerically (FEM) calculated loading curves of the elastic contact.

The application of Eq. 1 (${E}_{r}=190\,{\rm{GPa}}$ and $R=500\,{\rm{nm}}$) and supplementary finite element method (FEM) simulations show that the initial contact can be described as elastic contact between a spherical indenter and an elastic half-space (Fig. 4). During loading, the shear stress in a small subsurface volume increases until it reaches a critical value at which dislocation slip is activated, either by mobile preexisting dislocations or by dislocation nucleation. In particular, dislocation nucleation can cause a pronounced pop-in, with a sudden displacement burst resulting in a first pop-in. Most of the measured curves show at least one early pop-in, indicating that dislocation activation and dislocation nucleation play a decisive role. These findings are well supported by simulations^12,13^ and experimental investigations of other metallic materials^6,10,14^. Classical MD simulations showed that as the shear stress in a defect-free volume under the indenter tip reaches a critical value, homogeneous nucleation of a dislocation loop can occur^13^.

**Figure 5:**
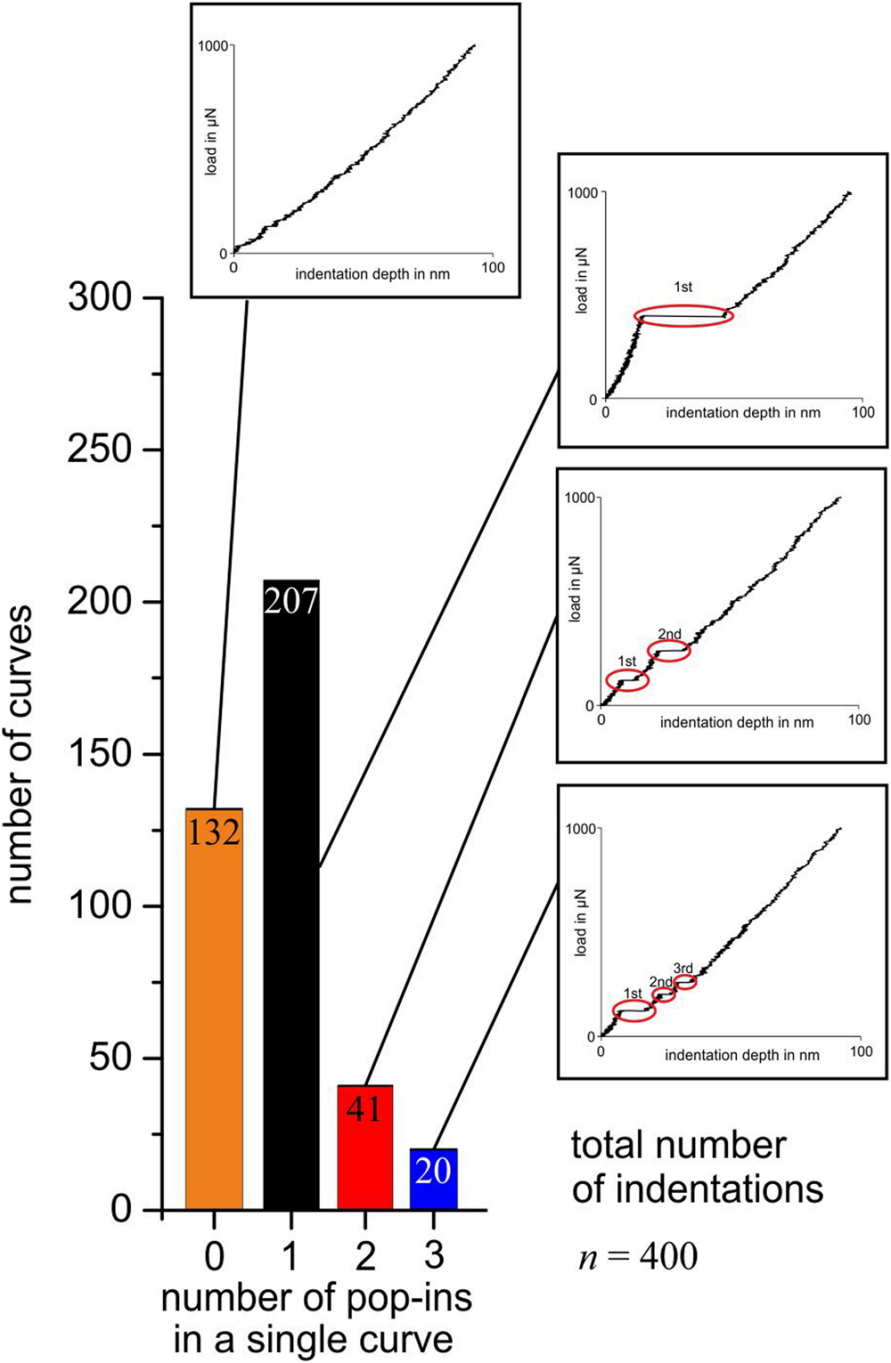
Number of observed pop-ins in 400 load–displacement curves measured in polycrystalline iron.

Figure 5 shows that two-thirds (268 of 400) of all the measured load–displacement curves have at least one pop-in. The maximum number of pop-ins in one curve is three (in the investigated load range). The critical load for pop-in initiation and the length of the displacement burst were quantified for all the curves. Pop-in initiation requires a load of 50 to 650 *μ*N. The length of the pop-ins varies between a few nanometers and 50 nm.

**Figure 6:**
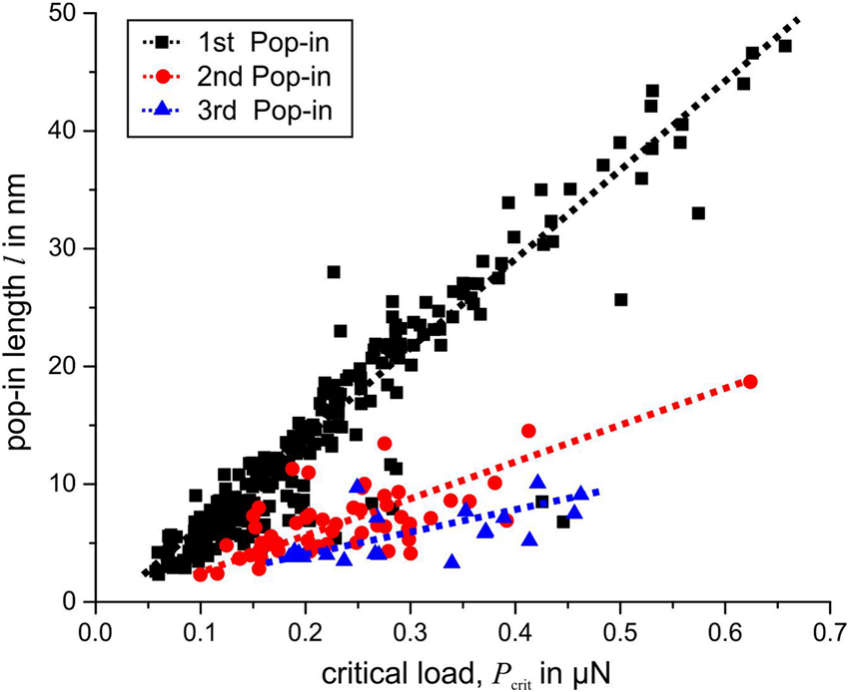
Correlation between pop-in initiation load and length of pop-in.

**Figure 7:**
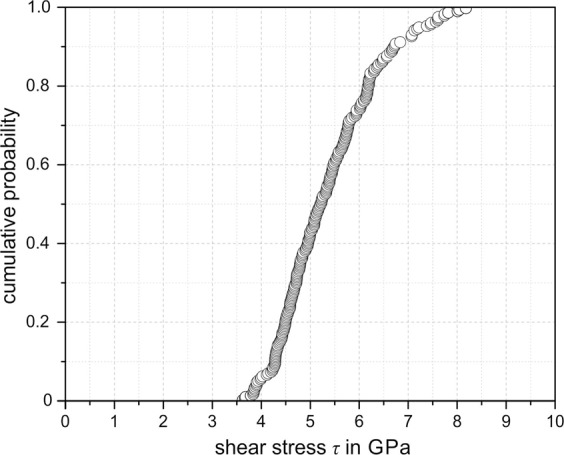
Cumulative probability of calculated shear stress for a first pop-in.

As Fig. 6 illustrates, there is a correlation between the critical load *P*_crit_ and the length *l* of each pop-in. With increasing load, the pop-in length increases more or less linearly. For a second or even third pop-in, the pop-in length for a given critical load is reduced. However, the fair linear correlation between the initiation load and pop-in length is maintained. Assuming an elastic-to-plastic transition for the first pop-in, Eq. 3 can be used to calculate the shear stress $\tau$ for a given critical load. The calculated shear stress varies between 3.5 and 8.5 GPa for the measured first pop-ins. Figure 7 shows the cumulative probability of the shear stress for a first pop-in. Ma *et al*.^15^ showed that the local indenter geometry and tip radius are crucial factors for determining the shear stress using the Hertz method. Irregularities in the local tip geometry and a large tip radius can cause higher values of the determined local shear stresses. For this reason, the shear stresses in Fig. 7 do not represent the critical shear stress for homogeneous dislocation nucleation with absolute precision. However, the results are in good agreement with the theoretical strength of a defect-free iron crystal, as discussed below.

**Figure 8:**
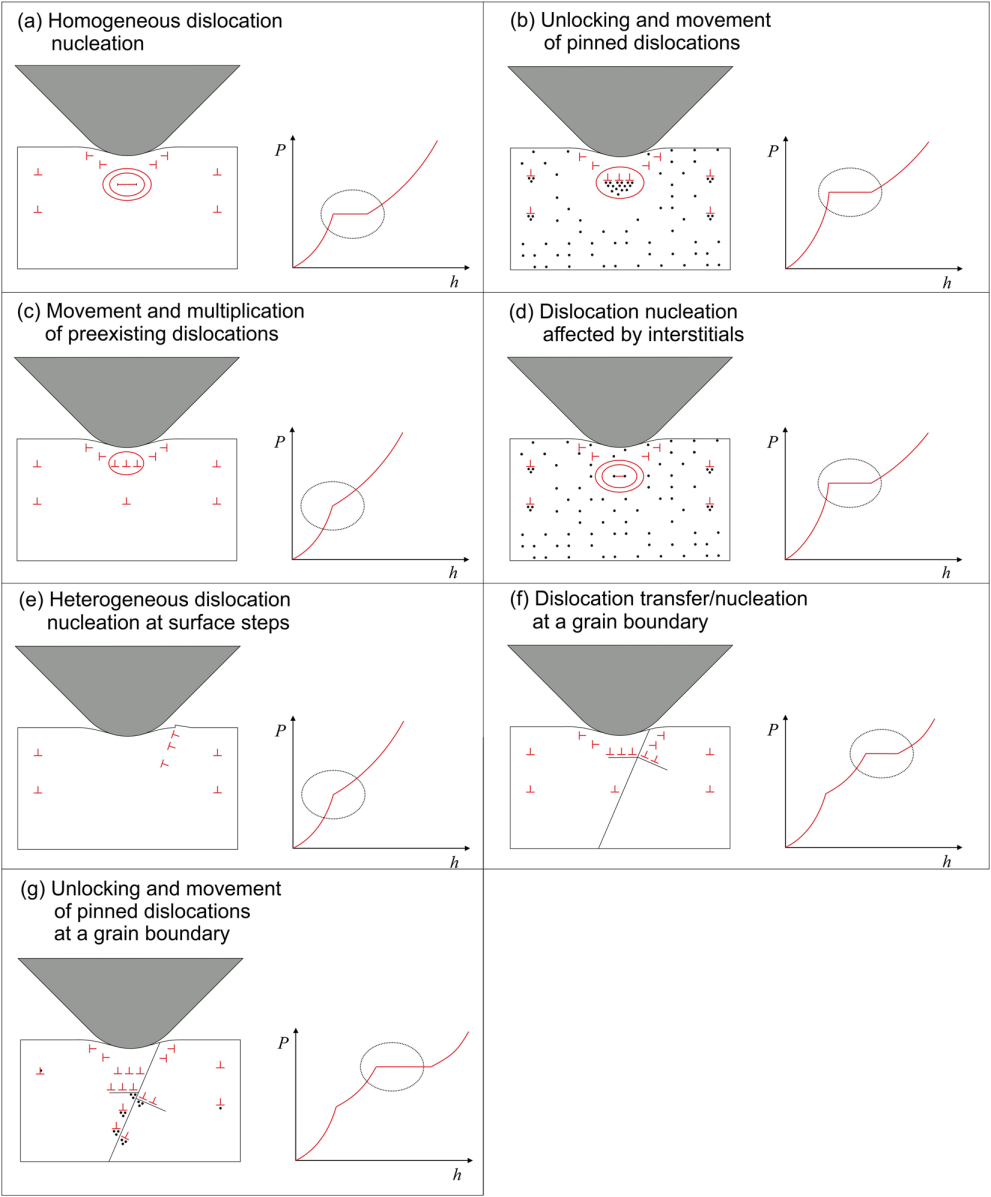
Schematic illustration of (**a**) homogeneous dislocation nucleation in a defect- and dislocation-free volume, (**b**) activation and unlocking of preexisting dislocations pinned by interstitials, (**c**) movement and multiplication of preexisting unpinned dislocations, (**d**) dislocation nucleation in a dislocation-free volume affected by interstitials, (**e**) heterogeneous dislocation nucleation on the surface at local surface steps, (**f**) dislocation transfer/nucleation at grain boundaries, and (**g**) activation and unlocking of preexisting dislocations pinned by interstitials at a grain boundary.

Figure 8 illustrates seven possible scenarios for the origin and mechanism of pop-ins. As a result of very careful specimen preparation, it is assumed that the initial stress field of the indenter is likely to include a dislocation- and defect-free volume. This situation is shown schematically in Fig. 8(a). For plasticity initiation, the shear stress has to reach a critical value at which homogeneous dislocation nucleation occurs. Ahn *et al*. measured shear stresses of 3–5 GPa for the first pop-in of a ferritic steel and concluded that the high shear stresses are a result of dislocation nucleation^10^. The shear stresses calculated in this study are in a very similar range, which is also of the same order as the theoretical strength of the material. The theoretical strength is approximately $\frac{1}{25}$ to $\frac{1}{15}$ of the shear modulus *G*. The shear modulus of pure iron is 80.7 GPa; thus, the theoretical strength is 3–5 GPa^16^.

Ahn also showed that the pop-in behavior of ferritic steels is influenced by dissolved interstitials such as carbon^10^. According to the literature, the dislocation density usually has a strong effect on the occurrence of pop-ins^7,9^. For a high dislocation density and preexisting mobile dislocations in the shear zone under the indenter tip, no distinct pop-ins are found because lower resolved shear stresses are necessary for dislocation activation and multiplication than for the homogeneous dislocation nucleation mentioned above. It is known that in pure iron and bcc steels, the mobility of preexisting dislocations can be influenced by interstitial atoms such as carbon. Dissolved carbon atoms can diffuse into the stress fields of preexisting dislocations to form Cottrell atmospheres, which cause pinning of dislocations. During nanoindentation, the pinned dislocations can be unlocked by a higher resolved shear stress with subsequent dislocation movement and multiplication, resulting in a sudden increase of indentation depth and a pop-in. Hence, the critical resolved shear stress might be significantly increased by the presence of Cottrell atmospheres. However, once the critical resolved shear stress is exceeded and pinned dislocations are released, lower resolved shear stresses are necessary for their movement and multiplication. Consequently, a sudden increase of indentation depth is observed under a constant load in a load-controlled indentation experiment. This situation is schematically illustrated in Fig. 8(b). Here, prestraining of the material would unlock dislocations and increase the dislocation density before the indentation test. Hence, pop-ins due to homogeneous dislocation nucleation of a defect-free volume and due to the unlocking of pinned dislocations should typically be avoided.

**Figure 9:**
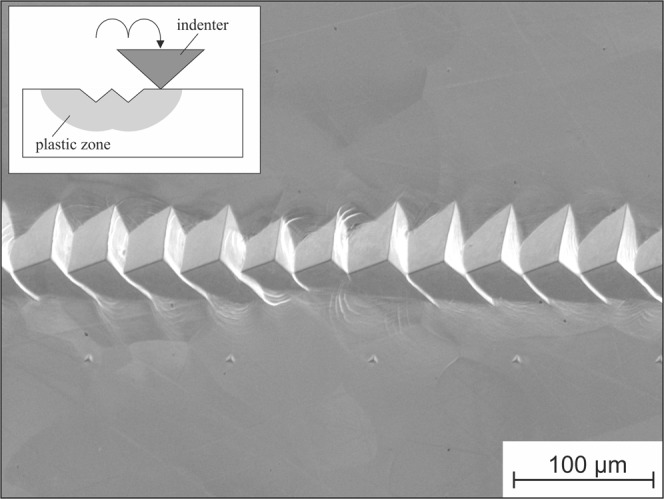
Nanoindentations made in the plastically deformed volume of previous indentations.

**Figure 10:**
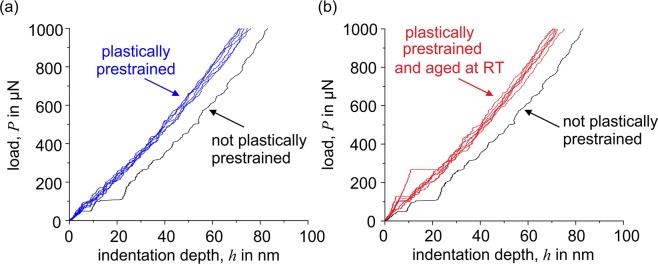
Load–displacement curves of nanoindentations made in the plastically deformed volume resulting from prior indentation. Curves were obtained (**a**) immediately after the second indentation and (**b**) after several weeks of aging.

**Figure 11:**
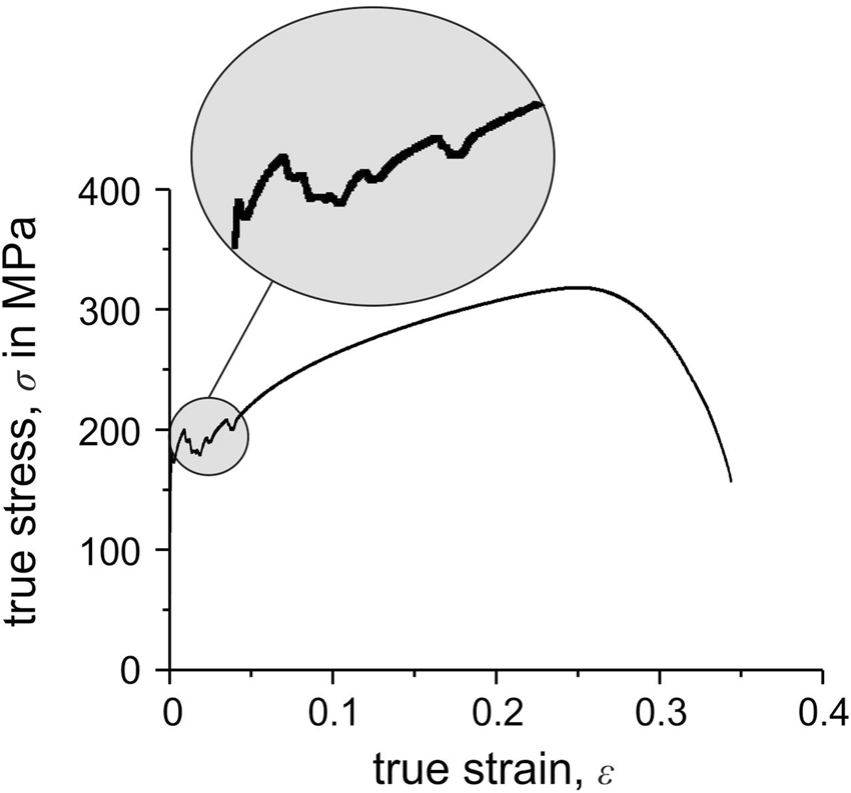
Stress–strain curve (tensile test) of the pure iron, which shows the appearance of Lüders strain.

Figure 9 shows nanoindentations that were made within the plastically deformed volume caused by a previous indentation. As Fig. 10(a) illustrates, most of the pop-ins disappeared. Only very isolated small pop-ins can still be identified. The same behavior caused by prestraining has also been observed for a ferritic steel^10^. Thus, the presence of unpinned mobile dislocations results in the disappearance of pop-ins. As shown in Fig. 10(b), aging the strain-hardened specimen at room temperature (RT) for several weeks results in the re-appearance of pop-ins. During aging, interstitial carbon atoms diffuse into the stress field of the introduced dislocations; consequently, they are again pinned. This demonstrates that pop-in is caused not only by dislocation nucleation but also by the unlocking of preexisting dislocations that are pinned by interstitials. A similar effect of interstitials is observed as the appearance of Lüders strain during a macroscopic tensile test^17^. As shown in Fig. 11, the investigated material also shows a significant macroscopic Lüders strain during tensile testing. This demonstrates that the phenomena are closely related, although they are analyzed on very different length scales. Here, the significant effect of even small quantities of interstitials on the deformation behavior and strength is clearly seen. In this context, the strong contribution of interstitials to the strength and the complex interactions between them and dislocations becomes apparent. Another effect of interstitial atoms on plasticity initiation was investigated by Sekido *et al*., who showed that not only do interstitials influence preexisting dislocations, but it is also very likely that they affect dislocation nucleation by decreasing the length of a Frank–Read source^6^. This situation is shown schematically in Fig. 8(d).

**Figure 12:**
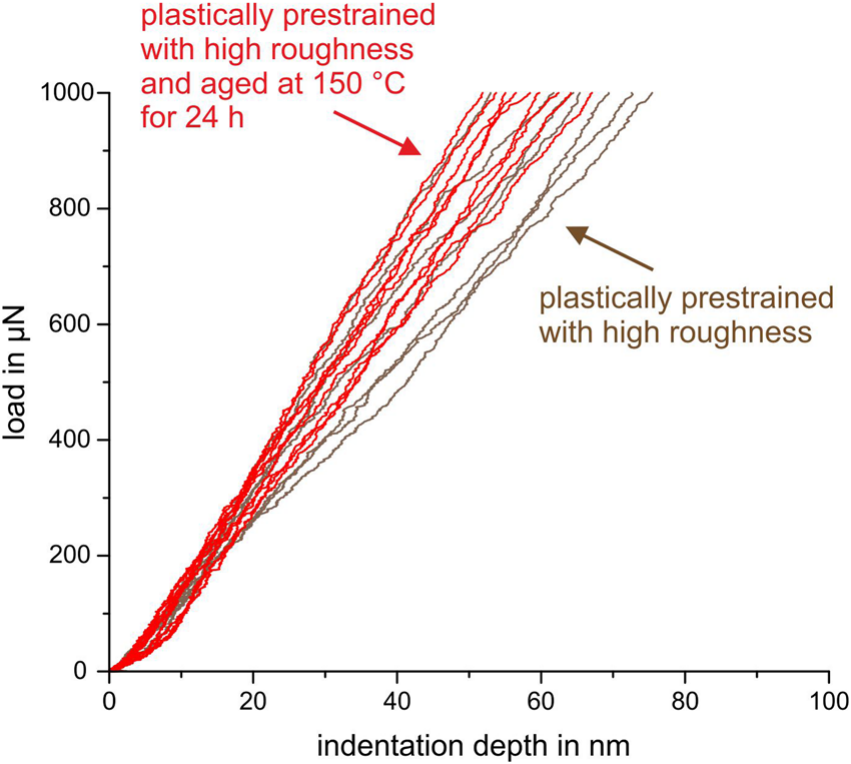
Load–displacement curves measured after plastic prestraining by rough surface finishing with 6 *μ*m diamond suspension and after an additional aging step at 150 °C for 24 hours.

For dislocation nucleation or activation and multiplication within a given volume under the specimen surface, roughness can also be a potential origin of dislocation nucleation. To investigate this possibility, we polished the specimen to a relatively rough surface finish using a diamond suspension with an average grain size of 6 *μ*m. As shown in Fig. 12, only very isolated small pop-ins were observed even after the sample was aged at 150 °C for 24 hours. This finding indicates that dislocations were nucleated from the surface or that mobile preexisting dislocations were present. Local stress concentrations caused by surface steps also favor the nucleation or activation of preexisting dislocations, leading to the disappearance of pop-ins. Beake and Goel also showed that at a high surface roughness, the number of pop-ins in tungsten is significantly reduced^18^. However, in their study, it was not possible to clarify whether the roughness itself or introduced defects are dominant in reducing the pop-in tendency. The results presented here suggest that the surface roughness itself plays a dominant role, because pop-ins were suppressed even after the aging and diffusion of carbon into the stress fields of the introduced dislocations. This illustrates that the surface condition and quality strongly affect the pop-in behavior. In addition, it is also apparent that surface roughness increases the scatter of the load–displacement curves for the investigated indentation depths. The nucleation of dislocations at areas of peak surface roughness is schematically illustrated in Fig. 8(e).

**Figure 13:**
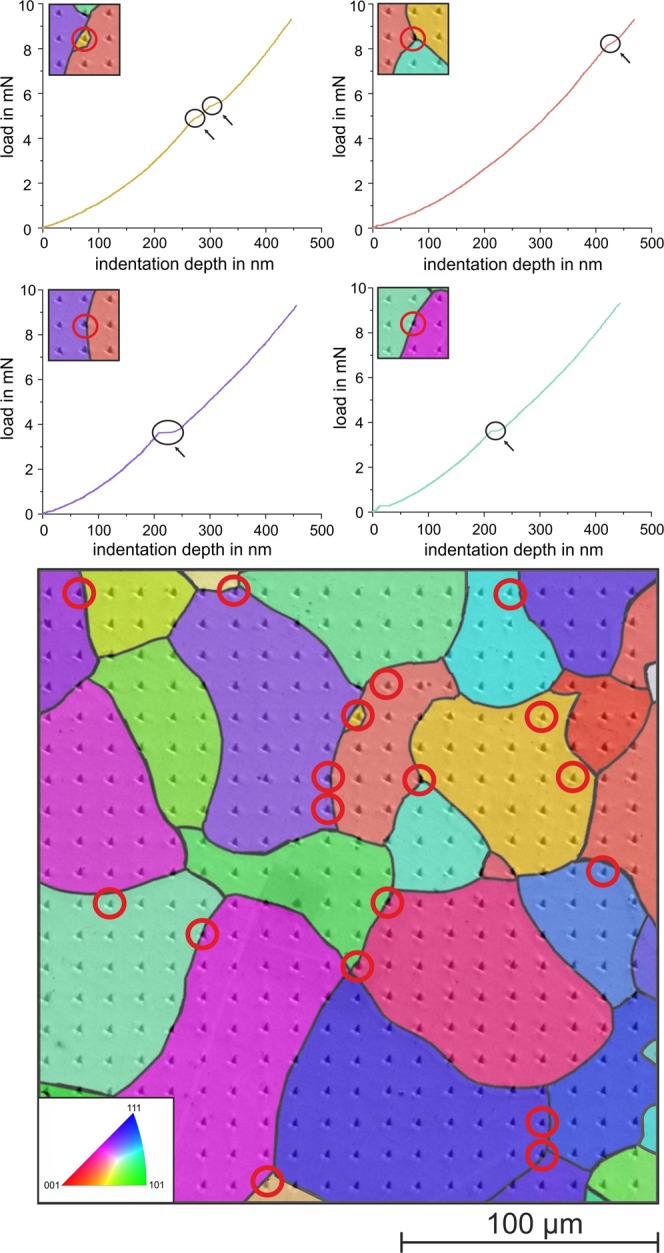
Location of all load–displacement curves that have a significant pop-in at high normal loads (red circles) and selected loading curves showing the pop-ins. All indentations with distinct pop-ins at high loads are near grain boundaries.

Another possible origin of pop-ins is the interaction of dislocations with a grain boundary. As shown in 8(f), dislocation slip and accumulation at a grain boundary can lead to dislocation transfer into or dislocation nucleation within a neighboring grain with a subsequent displacement burst. Several authors observed pop-ins resulting from such behavior^19,20^. Soer and De Hosson show for a Fe-14 wt.% Si alloy that Berkovich nanoindentation near a grain boundary leads to dislocation pile-up and transmission across the boundary. The latter mechanism can result in a pop-in in the *P*-*h* curve^21,22^. Shen *et al*. even argued that carbon segregation at grain boundaries during aging of a low-carbon steel could result in pronounced grain boundary pop-ins^20^. Thus, these pop-ins should also occur at relatively high loads, because their occurrence results from the stress field coming into contact with a grain boundary. The volume of the stress field is a function of the indentation depth and increases with the cube of the indentation depth^23^. Because the polycrystalline iron studied here has relatively large grains, it is assumed that this mechanism is rarely seen here, and only a few indentations out of 400 measurements are near or directly on a grain boundary with their stress field interacting with the boundary. However, 18 load–displacement curves have very pronounced pop-ins at relatively high loads. Figure 13 shows the positions of all 18 indentations in the microstructure and selected loading curves of these indentations. It is obvious that all the indentations are near grain boundaries. Hence, pronounced pop-ins at high loads are closely related to the presence of a grain boundary in the stress zone of the indentation. Although all the pop-ins at high loads can be allocated to indentations close to grain boundaries, not all the indentations in close proximity to a grain boundary have a pop-in at large loads. In general, as discussed above, a grain boundary can cause stress concentrations that result in slip transfer or dislocation nucleation in a neighboring grain, resulting in a pop-in. Regarding this effect, Britton *et al*. also concluded that interstitial atoms can diffuse to dislocations at the grain boundaries during aging, resulting in pinning of the grain boundary dislocations^24^. As noted above, pinning of dislocations can cause pop-ins. This mechanism is shown in Fig. 8 as (g).

**Figure 14:**
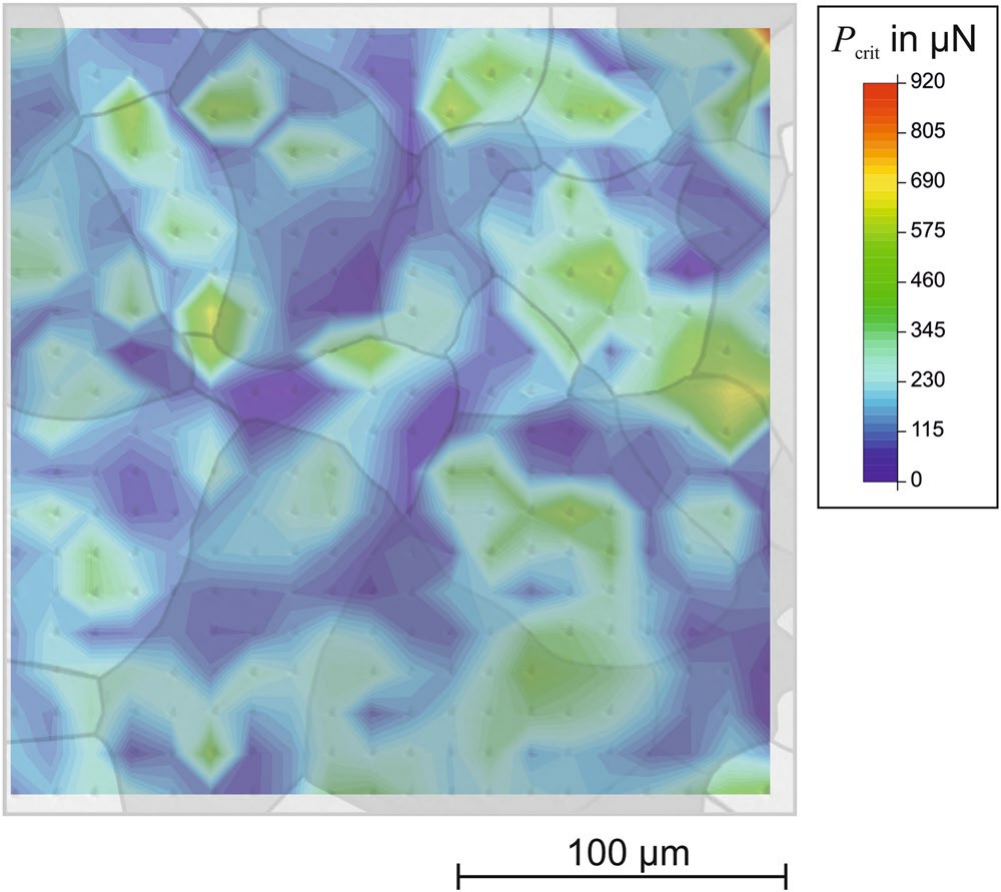
Interpolated distribution of critical load *P*_crit_ for the initiation of a first pop-in (analyzed loading range: 0–1000 *μ*N), revealing no correlation with the grain orientation.

Because the critical resolved shear stress in a glide system depends on the crystallographic orientation, one could assume that the critical load for the initiation of a first pop-in and the calculated shear stress should also be orientation-dependent. However, we found no correlation between the occurrence of pop-ins and the position of the indentations, which indicates that the crystallographic grain orientation had no observable effect. As an example, Fig. 14 shows the interpolated distribution of the critical load for the initiation of a first pop-in. As the figure demonstrates, no correlation with the grain orientation is found. Salehinia *et al*. used MD simulations to numerically investigate the effect of crystallographic orientation (in fcc metals) on dislocation nucleation and multiplication in the presence of lattice defects and showed that even small defects strongly affected the pop-in load^25^. Hence, the pop-in phenomenon can have numerous origins with complex interacting mechanisms, and its occurrence depends strongly on crystal defects, surface roughness, or local indenter geometry. These aspects are also statistically to a certain extend. Thus, observing a clear correlation between crystallographic orientation and pop-in occurrence is difficult.

Generally, the occurrence of pop-ins appears as a statistical process, whereas the previously discussed mechanisms can occur in isolation or in combination. The statistical nature also becomes apparent from the cumulative distribution of the shear stress for pop-in initiation (Fig. 7). As a function of dislocation density, interstitial distribution, local surface roughness, grain size, and grain orientation dependence, no or numerous pop-ins at different critical loads were observed within a single load–displacement curve. Multiple pop-ins might result from a combination of different mechanisms. For example, a first pop-in could be caused by dislocation nucleation, and a second pop-in could result from unlocking of preexisting pinned dislocations. Pronounced pop-ins at relatively high loads are likely to be caused by interaction between the stress field and a grain boundary, in combination with pinned dislocations. The individual mechanisms cannot easily be separated; thus, the pop-in behavior remains a highly statistical process, and experiments have not shown a statistically relevant dependence on the crystallographic orientation.

## Summary and Conclusions

In this study, we investigated the pop-in behavior of polycrystalline iron during nanoindentation. These investigations yielded the following conclusions:

- The analysis of several hundred indentations in polycrystalline iron showed the occurrence of zero, one, two, or even more pop-ins within a single load–displacement curve. Elastic analyses based on the Hertz method and FEM simulations revealed that the first pop-in is typically caused by plasticity initiation and thus by the elastic-to-plastic transition. The calculated critical shear stresses of 3.5 to 8.5 GPa are on the order of the theoretical strength and indicate homogeneous dislocation nucleation.
- Increasing the dislocation density by plastic deformation before the indentation experiment results in an absence of pop-ins. Indentation of a volume with a high dislocation density causes movement and multiplication of preexisting mobile dislocations rather than dislocation nucleation. The transition between initially elastic behavior and additional plastic behavior is continuous without a pop-in. Surface roughness can also suppress the occurrence of pop-ins with a continuous elastic-to-plastic transition due to activation of preexisting dislocations and heterogeneous dislocation nucleation at surface steps.
- Interstitial atoms such as carbon influence pop-in behavior by blocking preexisting dislocations, very much like the Lüders strain obtained in a macroscopic tensile test. The unlocking of dislocations pinned by carbon atoms (Cottrell atmospheres) can also result in a pop-in. Plastic deformation before the indentation experiment unlocks dislocations from interstitials and generally suppresses pop-ins, whereas subsequent aging causes them to reappear.
- Grain boundaries in the stress field of the indenter can cause pronounced pop-ins even at relatively high indentation loads. Dominant mechanisms such as slip transfer, dislocation nucleation in a neighboring grain, and the unlocking of pinned dislocations by carbon atoms at the grain boundary were considered.
- The occurrence of pop-ins has numerous different causes that cannot easily be separated from each other. Surface roughness, grain boundaries, grain orientation relationships, interstitial distribution, and dislocation density affect pop-in behavior, whereas no dependency of the crystallographic orientation on the pop-in behavior could be observed. The pop-in behavior of pure iron appears to be a statistical process governed by the mechanisms mentioned above.
